# Frequency and Correlation of Common Genes Copy Number Alterations in Childhood Acute Lymphoblastic Leukemia with Prognosis

**DOI:** 10.31557/APJCP.2020.21.12.3493

**Published:** 2020-12

**Authors:** Abbasali Hosein Pour Feizi, Sirous Zeinali, Jacek Toporski, Roghayeh Sheervalilou, Sahar Mehranfar

**Affiliations:** 1 *Hematology and Oncology Research Center, Tabriz University of Medical Sciences, Tabriz, Iran. *; 2 *Department of Molecular Medicine, Biotechnology Research Center, Pasteur Institute of Iran, Tehran, Iran. *; 3 *Kawsar Human Genetics Research Center, Kawsar Genomics Center, Tehran, Iran. *; 4 *Department of Clinical Sciences, Pediatric Oncology and Hematology, University of Lund, Lund, Sweden. *; 5 *Pharmacology Research Center, Zahedan University of Medical Sciences, Zahedan, Iran. *; 6 *Department of Genetics and Immunology, Faculty of Medicine, Urmia University of Medical Sciences, Urmia, Iran. *; 7 *Social Determinate of Health Research Center, Clinical Research Institute Urmia University of Medical Science, Urmia, Iran. *

**Keywords:** Childhood acute lymphoblastic leukemia (ALL), Multiplex ligation, dependent probe amplification (MLPA)

## Abstract

**Objective:**

It was shown by genomic profiling that despite no detectable chromosomal abnormalities a proportion of children with pre-B acute lymphoblastic leukemia harbors copy number alterations (CNA) of genes playing role in B-cell development and function. The aim of the study was to determine the frequency of CNA in pediatric acute lymphoblastic leukemia and correlate these findings with clinical outcome.

**Methods:**

DNA extracted from peripheral blood or bone marrow at diagnosis/relapse of fifty newly diagnosed children with precursor B-cell acute lymphoblastic leukemia was analyzed for CNA with multiplex ligation-dependent probe amplification.

**Results:**

The analysis revealed 76 CNA in 24 patients most frequently found in *PAR1* (17%), *CDKN2A/B* (15.7%) and *PAX5* (14.4%) genes. There were significant CNA co-occurrences between *PAX5, CDKN2A/B, BTG1, ETV6, PAR1 *or *XP22* genes, (p<0.020) and the high-risk group. There was a significant correlation between* EBF1, RB1,* and* IKZF1* alterations and bone marrow relapse. Patients with CNA in screened genes are more likely to succumb to their disease except for those with *PAR1 *or *XP22* genes (p<0.050).

**Conclusion:**

The multiplex ligation-dependent probe amplification could be considered as an independent diagnostic tool allowing prompt identification of patients at high risk of treatment failure and, subsequently, a more adequate treatment approach.

## Introduction

Acute lymphoblastic leukemia (ALL) is the most common childhood malignancy. About 75% of children with leukemia have various chromosomal abnormalities. Although 85-90% of child patients are cured with modern therapies, relapses occur in approximately 15-20% of cases. Previous studies showed that treatment failure is strongly related to the site and timing of relapse (Bailey et al., 2008; Parker et al., 2010; Moorman, 2012; Yamashita et al., 2013). Also, other studies revealed that the cells existing in the relapse clone, were often present at the time of first diagnosis (Reismüller et al., 2009; Vardiman, 2010). These data suggest that we can determine the risk of relapse by screening common involved genes before chemotherapy and during treatment and also on follow-up (Mullighan et al., 2008a). The risk of specific chromosomal abnormalities that are important for diagnosis and treatment are well-documented (Harrison et al., 2010; Harrison and Johansson, 2015). In about 20-25% of cases with normal karyotype, further investigation is required to determine the molecular factors affecting the prognosis (Pui et al., 2004; Pieters and Carroll, 2008). Molecular studies have indicated that copy number abnormality (CNAs) of these genes play a central role in the pathobiology of lymphoid leukemia. Many of these alterations have an initiating role in leukemogenesis pathway and they have a significant impact on prognosis and therapeutic decisions (Kuiper et al., 2007; Mullighan et al., 2007; Strefford et al., 2007; Zhou et al., 2012). Previous studies have identified numerous deletions in signaling pathways of precursor B-cell (BCP) differentiation (IKZF1, ETV6, PAX5, and EBF1), cell cycle control, tumor suppression (CDKN2A/2B, BTG1, and RB1), and cytokine receptors (*CRLF2, IL3RA, CSF2RA, *and *SHOX*) localized in the pseudoautosomal region (PAR1) (Kuiper et al., 2007; Mullighan et al., 2007). 

*CNAs *of *IKZF1* and the most famous genes of *PAR1 *region have been shown to be associated with poor prognosis in BCP-ALL (Mullighan et al., 2009b; Russell et al., 2009; Schwab et al., 2010; Yang et al., 2011). Ma et al. in 2015 showed that half of cases of leukemia had multiple sub-clonal mutations in a pathway or genes at diagnosis but mostly with only one minor clone. Their results provide important insights into the genetic basis of chemotherapy failure in ALL (Ma et al., 2015). Single nucleotide polymorphism (SNP), array-based comparative genomic hybridization (aCGH) and fluorescence in situ hybridization (FISH) have been used previously for detection of these abnormalities, but all of them had important disadvantages such as being expensive, labor-intensive and unavailable in most of the labs. Consequently, the aforementioned causes make these methods inapplicable for routine diagnosis or screening large cohorts (Harrison et al., 2005; Coll-Mulet et al., 2008; Russell et al., 2009; Stevens-Kroef et al., 2009; Al Zaabi et al., 2010). Therefore, it is necessary to ﬁnd a rapid and reliable method for screening these abnormalities to determine their incidence, interrelationship and prognostic signiﬁcance. Multiplex ligation-dependent probe amplification (MLPA) is a rapid, low-cost and simple multiplex polymerase chain reaction (PCR)-based technique introduced by Schouten in 2002 (Schouten et al., 2002). It can distinguish about 50 different genomic lengths of DNA or RNA sequences in one reaction (www.MRC-Holland.com). 

This study has used MLPA method for the simultaneous screening of these abnormalities, applicable to large cohorts of patients with BCP- ALL. A variety of mutations occur more frequently in certain races and ethnic groups, such as CRLF2 in Hispanics (Mullighan et al., 2007; Familiades et al., 2009; Nebral et al., 2009; Harvey et al., 2010). Thus, inherited genetic variations are important in the pathogenesis of ALL (Hunger and Mullighan, 2015). There is no record of CNAs in Iranian ALL patients. Therefore, this study was done to find the prevalence of CNAs in our BCP-ALL patients.

## Materials and Methods

*Patient selections*

**Figure 1:**
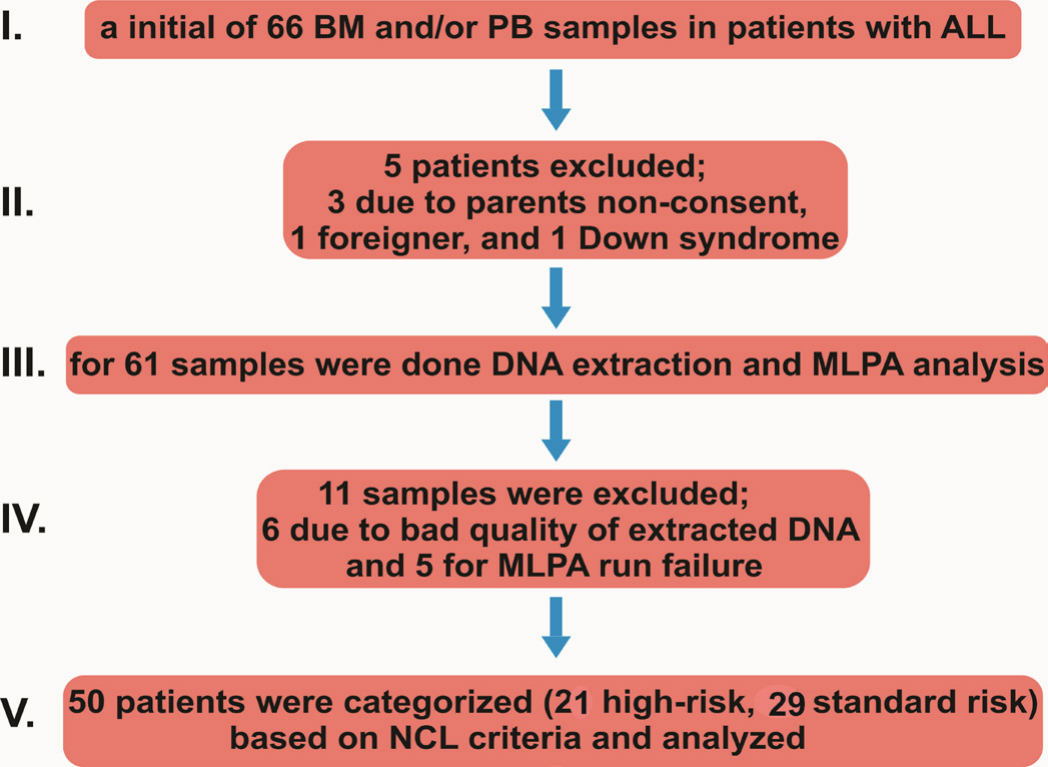
The Sampling Process

Patients with BCP-ALL registered with Children’s Hospital of Tabriz Medical Sciences University (TBZMED) (2014-2016) were eligible for this study (Table1). They were diagnosed by standard criteria. The inclusion criteria embraced the patients with proven ALL, and being 1-14 years age at the time of first diagnosis or first BM relapse before any salvage therapy. Exclusion criteria embraced the acute leukemia in children with Down syndrome and samples from foreigners. Initially 66 BM and/or peripheral blood (PB) samples were obtained from untreated patients with ALL. All patients were follow up for one year. 21 Patients were categorized as high-risk (HR) or standard risk based on the National Cancer Institute (NCI) criteria. High-risk category included WBC count≥50x109/ml and/or age≥10 years. Standard risk (SR) category included, WBC count≤50 x 10^9^/ml and/or age between 1-10 years old (Mullighan et al., 2009b) (Figure 1).

*Sample selection*

DNA samples were obtained freshly from PB, BM or both. Additionally, 5 BM and 10 PB samples from non-malignant patients matched the samples from control group in terms of age and sex.

*DNA extraction*

DNA extraction was done by using the QIAamp DNA minikit (Qiagen, Hilden, Germany). The final DNA concentration was determined by NanoDrop spectrophotometry (NanoDrop, Wilmington, DE, USA) and the purity was assessed by 2% agarose gel electrophoresis.

*Definition of P335-B2 SALSA MLPA kit*

DNA obtained from samples was used to determine the CNAs of IKZF1, CDKN2A/B, PAX5, EBF1, ETV6, BTG1, RB1, and PAR1 region , using the P335-B2 SALSA MLPA kit (MRC-Holland, Amsterdam, the Netherlands) (Schwab et al., 2010). Seven control samples were taken in each run of MLPA, to compare the patients’ with control peaks. Relative copy number was gained after normalization of peaks against control. The PCR fragments were separated by capillary electrophoresis on a 3130XL Genetic Analyzer (Life Technologies, Carlsbad, CA, USA). MLPA data were analyzed using GeneMarker V1.95 Soft Genetics (State College, Pennsylvania). The primer sequences for Pax5 have been presented in supplementary Table 1.

*Statistical Analysis*

Normality of the data was assessed by Kolmogorov-Smirnov test and expressed by the mean and standard deviation. Non-normal variables were implied as median (max-min). The gene *CNAs* difference between the ALL and the control samples were calculated by independent sample T-test. To assess the agreement between genes detected by MLPA, we used the kappa test. In order to determine the importance of quantitative factors in risk grouping, “independent variable importance” analysis which is a predictive test was employed. Also, to determine the association of genes with risk of relapse and mortality and their predictive importance, we used “contingency coefficient” test. Study variables were compared by the Chi-square or the Mann–Whitney test, for categorical and non-normal numeric variables respectively. P-values<0.05 were considered statistically significant. For the kappa test values, the level> 0.7 were considered strong agreement and <0.4 had a weak agreement. All statistical analyses were performed using the Statistical Package for the Social Sciences (SPSS 16.0, Inc, Chicago, IL, USA).

## Results

Sources of DNA samples were from PB in 30 (53.5%) and BM in 26 (46.5%) patients. In six patients, both BM and PB samples were taken simultaneously. Molecular analysis was carried out on 56 samples from 50 patients. All patients were follow up for one year. 21 patients (42%) were assigned to high-risk group and 29 of them (58%) to standard risk. 12 patients (24%) belonged to BM relapsed group, 5 of which originally high-risk BCP-ALL. 10 patients (20%) who died subsequently because of refractory diseases, lodged in mortality group. No significant difference was observed between sex, age and sample source in various groups, except a significant correlation between high WBC and high-risk group (p=0.001).

*MLPA findings*

Overall, 76 CNAs was found in 24 patients (48%), who harbored at least one up to eight deletions or duplications in the genes studied. Deletions were more frequent than duplications (59.3% versus 40.7%). Also, simultaneous aberrations were observed in different genes. Among all of the aberrations, four patients (16.6%) had CNA in one, six (25%) had in 2, nine (37.5%) had in 3- 4 and five (20%) had in 5-7 genes. One patient, who had CNAs in all the genes under study, subsequently succumbed to refractory multiple relapses. Three most-prevalent CNAs in the order of frequency were XP22 (17%), CDKN2A/B (15.7%) and PAX5 (14.4%), whereas deletion of RB1 (7.8%) was the least-frequent (Table2). 

*Relationship between MLPA results with WBC count*

We found a statistically significant relationship between CDKN2A/B and having a high initial WBC count in patients with ALL (p=0.03). Also, there was a considerable, yet not significant relationship between PAX5 and WBC (p=0.08). Three or more deletions were signiﬁcantly more frequent in adult children (p=0.04) and in those patients with a high WBC count (p=0.03). Data was shown in supplementary Table 2.

*Relationship between MLPA findings and risk parameters*

**Figure 2:**
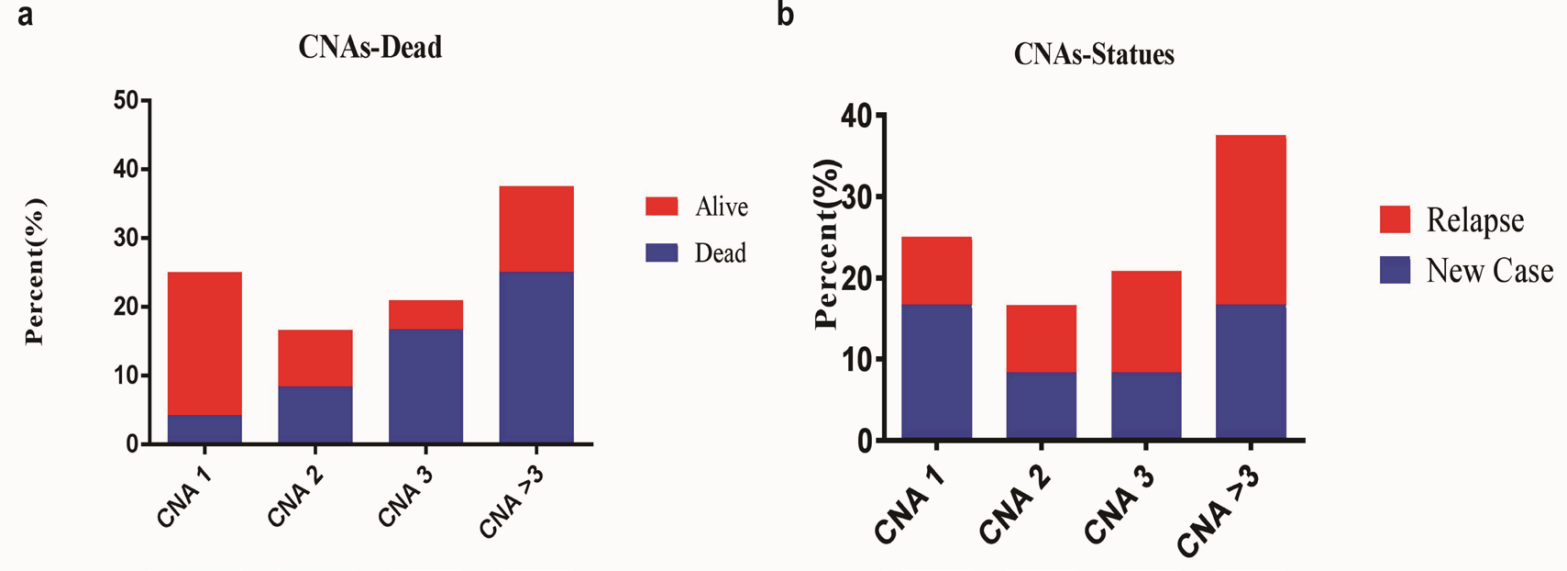
The Evaluation of Correlation between CANs with a) the death in high-risk group, b) the patients relapse. Results showed a significant concurrency between *CNAs* of *PAX5, ETV6,* *CDKN2A/B* and *PAR1 *region genes, and being in high-risk group (2a), and a strong correlation between *EBF1, RB1* and *IKZF1 *and *BM* relapse group (2b)

We found a significant correlation between *CNAs *of *PAX5, CDKN2A/B, ETV6, PAR1* region genes (all p-values<0.020) and being at high-risk group. There was also a strong correlation between *EBF1, RB1*, and *IKZF1* (p=0.0001, 0.018 and 0.050, respectively) and *BM* relapse group. Patients with CNAs in all interesting genes are more likely to succumb to their disease except *PAR1* region genes (p-values<0.050). There was a significant concurrency between *CNAs* of *PAX5, ETV6, CDKN2A/B* and* PAR1* region genes, and being in high-risk group (Figure 2a). Also, we found strong correlation between *EBF1, RB1* and *IKZF1 *and *BM* relapse group (Figure 2b). Except for *PAR1* region genes, patients with all genes *CNAs* are more likely to succumb to their disease. Patients classified as high-risk group had a greater number of *CNAs *compared to standard risk group (p<0.05). *CNAs* were increasing with age in most of the studied genes. Data was shown in supplementary Table 2.

*The most common involved genes exons*

Whole gene deletion in *IKZF1* was observed in 40%, of whom 60% was restricted to exons 1 and 8. The majority of* PAX5 CNAs* in our study were in exons 6, 7and 8 (55.5%). Interestingly, we found a wide range of alteration in the *PAX5* gene. Regarding *ETV6*, the most involved exons were 1a, b and exon 5 (62.5%), and in one case all abnormalities were observed. Deletions of RB1 were the quite rare and they were present only in 8% of the cases. The most frequent exon was 26 (80%). In addition, deletions within PAR1 region were detected in 17% of patients. Duplications were more frequent than deletions. The most common duplications were found in *IL3RA* and *P2RY8* genes (Table 2).

*Co-occurrence of nine common genes aberrations*

**Figure 3:**
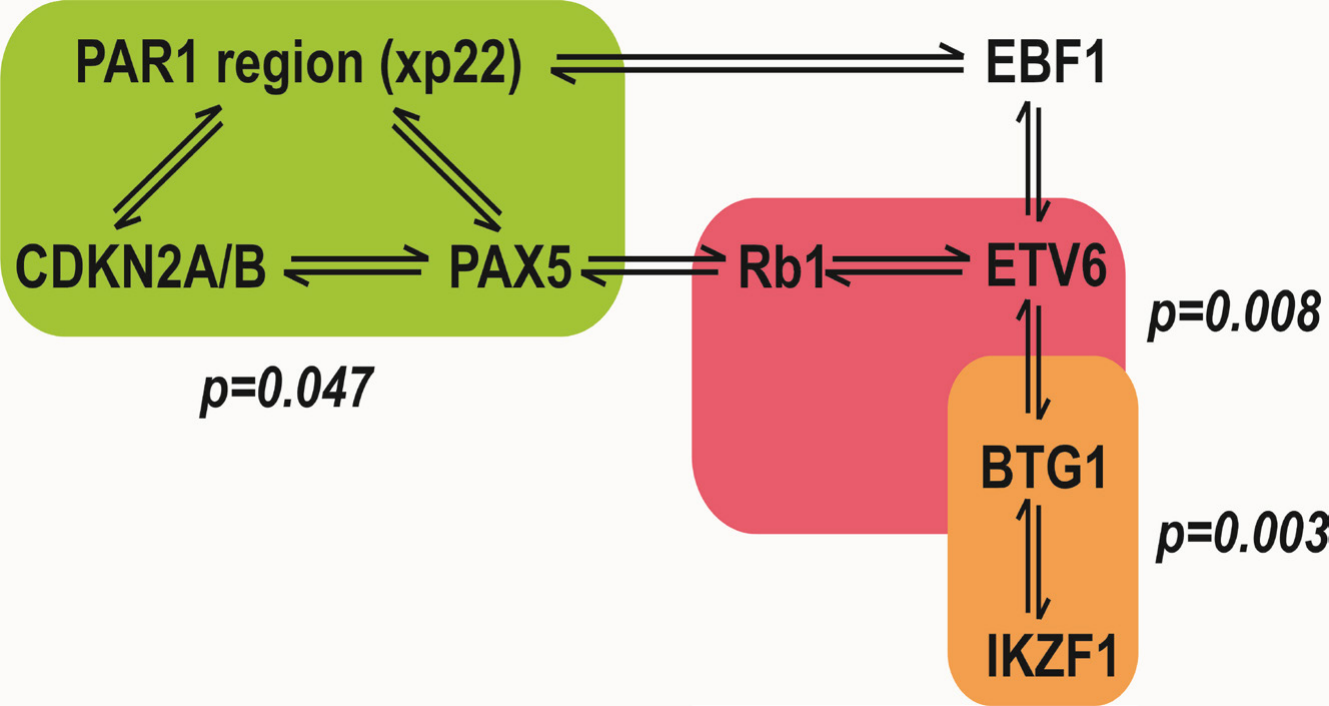
Schematic Correlation between Different Genes under Study Based on Kappa Test Results of Study. Results showed a novel coincidences between *CNAs* of the following genes: *CDKN2A/2B* and *PAX5; XP22* and *EBF1; XP22 *and *CDKN2A/B; XP22* and *PAX5* and* EBF1* with* ETV6, *and also, strong correlations between *ETV6* and *BTG1 *and *RB1*

There were novel coincidences between *CNAs* of the following genes: *CDKN2A/2B* and *PAX5* (kappa=0.59); *XP22* and *EBF1* (kappa=0.45); *XP22* and *CDKN2A/B *(kappa= 0.64);* XP22* and* PAX5* (kappa=0.59) and *EBF1* with *ETV6* (kappa=0.55). Also, Strong correlations were observed between *ETV6* and *BTG1* (kappa=0.8) and RB1 (kappa=0.55). The positive agreements between genes are presented schematically in Figure 3.

## Discussion

We studied the frequency of CNAs of common genes involved in development and differentiation in lymphoid cell (Supplementary Figure. 1) in children with *BCP-ALL* in Northwest of Iran. In our study the most frequent CNAs were firstly PAR1 region (17%, 10 Dup and 3 Del). PAR1 region contains at least 24 genes located in pseudoautosomal regions of X and Y chromosomes that are an important in hematopoietic development systems (Mullighan et al., 2008b; Mullighan et al., 2009a). 

*CNAs* of *CDKN2A/ B* (15.7%, 10DEL and 2DUP) were the second most frequent genes. These tumor suppressors inhibit *CDK4* kinase and have the regulatory role in controlling the cell cycle and progression (Martel et al., 1997) and their deletions are important in children with ALL. Mirebeau et al. (2006) demonstrated that patients with* CDKN2A/B* deletions have a shorter time to relapse than others (Yang et al., 2008; Krentz et al., 2013). In line with our results, some studies indicate an adverse effect on risk group (Fizzotti et al., 1995; Carter et al., 2001; Dalle et al., 2002). The prognostic impact of* CDKN2A/B *deletions in pediatric ALL is still controversial (ALLs, 2005; Novara et al., 2009; Sulong et al., 2009; Abdool et al., 2010; Sarhadi et al., 2013).

PAX5 was the third frequent occurring *CNAs* in our study. Ofverholm et al., (2013) in their study found *PAX5 *gene alteration second in rank among other genes, having the frequency of about 35% that is hallmark in relapse of disease, in childhood ALL patients. Our results revealed that this gene had an adverse effect on risk group. *PAX5 *and *EBF1* are transcription factors, which had a key role in STAT5 pathway that is important in differentiation, proliferation and survival (Heltemes-Harris et al., 2011b) and B-cells differentiations (Pongubala et al., 2008; Treiber et al., 2010). We conclude that EBF1 has more important role for determining relapse groups and mortality compared with PAX5 in these patients. 

As an anti-proliferative gene, *BTG1* affects cell cycle (G0 to G1) and apoptosis. van Galen et al., (2010) concluded that it plays a role in the sensitivity of glucocorticoid receptors, resistance to chemotherapy and increasing the mortality. Similar results were obtained in this study, although in high-risk group, it demonstrated considerable role, yet not significant.

*ETV6* is an oncogenic gene. Previous studies found ETV6 deletions in childhood ALL with normal karyotype (Bajaj et al., 2011). There are few studies with fewer subjects, which support the association of ETV6 deletions with poor outcome in adult ALL (Gomez-Segui et al., 2011; Moorman et al., 2012). Our data showed significant correlation between ETV6 and increase in risk of disease and mortality. 

*IKZF1 *is a tumor suppressor gene and a transcription factor regulator of lymphocyte. The isoforms of *IKZF1* differ in the number of N-terminal zinc finger motifs that bind DNA and are thought to function as dominant-negative factors. Deletions of exons 4-7 result in expression of dominant Negative IKZF1 isoform and shows oncogenic activity (Iacobucci et al., 2008). Loss of exon 8 will have an effect on dimerization of IKZF1, and results in inactivation, therefore it has the same impact as whole gene deletions (Mullighan et al., 2008a). Heltemes et al. showed that *IKZF1*and *PAX5 *were the most common mutations in their ALL patients (Heltemes-Harris et al., 2011a). In our study, deletion in exon 8 (60%) of* IKZF1* is common. 

Our data show that association between the number of CNAs and higher initial WBC is more important than age range in all of interesting genes. Moreover, about 75% of the patients had concurrent CNAs in interesting genes. The co-occurrence of *CDKN2A/B* and *PAX5 *genes in the mortality and high risk group were high 6 (25%), as illustrated by the previous studies conducted by others (Yang et al., 2008; Mullighan et al., 2009b; van der Veer et al., 2013). These patients often died before achieving remission with induction or re-induction chemotherapy. Besides, we showed the importance of co-occurrence between *PAX5* or *CDKN2A/2B* and *PAR1* region and between *ETV6, BTG1* and *IKZF1*. The clinical impact of alterations in *RB1, BTG1* and* ETV6* genes in childhood ALL have not yet been fully appreciated by other researchers (Moorman et al., 2010; Moorman et al., 2012; Schwab et al., 2013).

Similar to previous studies, our study also demonstrated that patients without any* CNAs*, were found in a standard risk group with better outcomes (Moorman et al., 2014; Barbosa et al., 2015). These finding indicate that each of these genes not only have a unique unfavorable effect on the disease process, but also may have a worse synergic effect on prognosis. We can hypothesize that their synergy effect on risk of disease and death are related to their linkage to the same chromosome (i.e.; *PAX5* and *CDKN2A/B* on chromosome 9). Indeed, Concurrent deletion of *PAX5* and *CDKN2A/2B* suggests that there are common targets in the pathogenesis of *BCP-ALL *(Kim et al., 2011; Schwab et al., 2013). One of the first antigens expressed on pro-B cells before transforming to blast is *CD19*, which is expressed by* PAX5*. On the other hand, the function of each gene could be modified by other genes in differentiation of blast cells.

These findings highlight the importance of identifying common genes in each population and their screening for identification of high risk patients. Our results are similar to other studies such as the findings of aforementioned studies but, there were some differences due to ethnic origin (Mullighan et al., 2007; Mullighan et al., 2008b; Moorman et al., 2012; Bhandari et al., 2017; Gupta et al., 2017).

It is obvious that increasing the frequency of mutations and involving larger loci of each gene have stronger effect on risk of disease and mortality. The correlation between these genes is important for decision on treatment plan and prediction of ALL prognosis (Gupta et al., 2017). Our results are in line with the findings of the comprehensive study conducted by Mullighan et al., (2008). However, there were some differences due to ethnic origin. 

This study demonstrated that the pattern of *CNAs *in following genes, are different. In addition, there is an association between* CNAs* of these genes and the risk of being grouped in the risk group. Differences in results in our population compared with other population can be explained by ethnic factors. Finally, our perception of the genetic mechanisms of relapse has improved significantly in the last few years, yet using this genetic knowledge and applying this information in clinical practices remain as important challenges. In addition, our results showed that MLPA is reliable method, appropriate for screening especially in countries with high prevalence of cancer and low income. However, due to the low number of patients, a study with a large number of patients is required to confirm or reject the results of this study.

**Table 1:** Demographic Data of Acute Lymphoblastic Leukemia Patients

Demographic data	N (frequency)	
Sex n (%)	Female	19 (38%)
	Male	31 (62%)
Age (years) n (%)	1-4	22 (44%)
	5-10	21 (42%)
	10-14	7 (14%)
Mean age (mean±SD)	Female	6.0 ±2.7
	Male	5.8±3.4
WBC count n (%)	<10	20 (40%)
	10-49.9	16 (32%)
	>50	14 (28%)
risk group n (%)	Standard risk	29 (58%)
	High-risk	21 (42%)

**Table 2:** Prevalence of Gene Copy Number Abnormalities by MLPA Method in Relapse, High-Risk and Mortalitygroups

Gene	CNA (frequency) (n=76 ) (%)	High-risk group (21 pts., 42%) P value*	Relapse Group (12 pts., 24%) P value*	Mortality group (10 pts., 20%) P value*	Common involved exons (%)
*XP22 OR PAR1*	13 (17)	0.002	0.325	0.325	Il3RA,P2Ry8,CRLF2
*CDKN2A/B*	12 (15.7)	0.001	0.181	0.04	5 (100), 2a (75)
*PAX5*	11 (14.4)	0.002	0.463	0.048	6, 7, 8 (55.5)
*ETV6*	10 (13)	0.002	0.1	0.048	1a, 1b (90), 5(62.5)
*IKZF1*	9 (11.8)	0.063	0.055	0.002	1 (40), 8 (60)
*BTG1*	8 (10)	0.068	0.1	0.043	1 (75), 2 (50)
*EBF1*	7 (9.2)	0.063	0.001	0.02	10 (71), 14 (71)
*RB1*	6 (7.8)	0.4	0.018	0.007	26 (80)
